# Could Digital PCR Be an Alternative as a Non-Invasive Prenatal Test for Trisomy 21: A Proof of Concept Study

**DOI:** 10.1371/journal.pone.0155009

**Published:** 2016-05-11

**Authors:** Laïla Allach El Khattabi, Christelle Rouillac-Le Sciellour, Dominique Le Tessier, Armelle Luscan, Audrey Coustier, Raphael Porcher, Rakia Bhouri, Juliette Nectoux, Valérie Sérazin, Thibaut Quibel, Laurent Mandelbrot, Vassilis Tsatsaris, François Vialard, Jean-Michel Dupont

**Affiliations:** 1 INSERM, U1016, Institut Cochin, CNRS UMR8104, Université Paris Descartes, Paris, France; 2 Laboratoire de Cytogénétique, Hôpital Cochin-Maternité Port-Royal, AP-HP, Paris, France; 3 Fédération de Génétique, CHI de Poissy St Germain, Poissy, France; 4 GIG, Faculté des Sciences de la Santé, Université de Versailles Saint-Quentin-en-Yvelines, Montigny le bretonneux, France; 5 Centre d’Epidémiologie Clinique, Hôtel Dieu, Paris, France; 6 Assistance Publique-Hôpitaux de Paris, Hôpital Cochin, Laboratoire de Biochimie et Génétique Moléculaire, Paris, France; 7 Département d’Obstétrique et de Gynécologie, CHI de Poissy St Germain, Poissy, France; 8 Service de Gynécologie-Obstétrique, Hôpital Louis Mourier, Hôpitaux Universitaires Paris-Nord Val de Seine, Colombes, France; Université Paris-Diderot, Paris, France; 9 Département d’Obstétrique et de Gynécologie, Hôpital Cochin-Maternité Port-Royal, Université Paris Descartes, Paris, France; Hospital Authority, CHINA

## Abstract

**Objective:**

NIPT for fetal aneuploidy by digital PCR has been hampered by the large number of PCR reactions needed to meet statistical requirements, preventing clinical application. Here, we designed an octoplex droplet digital PCR (ddPCR) assay which allows increasing the number of available targets and thus overcomes statistical obstacles.

**Method:**

After technical optimization of the multiplex PCR on mixtures of trisomic and euploid DNA, we performed a validation study on samples of plasma DNA from 213 pregnant women. Molecular counting of circulating cell-free DNA was performed using a mix of hydrolysis probes targeting chromosome 21 and a reference chromosome.

**Results:**

The results of our validation experiments showed that ddPCR detected trisomy 21 even when the sample’s trisomic DNA content is as low as 5%. In a validation study of plasma samples from 213 pregnant women, ddPCR discriminated clearly between the trisomy 21 and the euploidy groups.

**Conclusion:**

Our results demonstrate that digital PCR can meet the requirements for non-invasive prenatal testing of trisomy 21. This approach is technically simple, relatively cheap, easy to implement in a diagnostic setting and compatible with ethical concerns regarding access to nucleotide sequence information. These advantages make it a potential technique of choice for population-wide screening for trisomy 21 in pregnant women.

## Introduction

Screening for trisomy 21 is still mostly based on a risk estimation derived from the measurement of (i) biochemical markers in maternal blood and (ii) first trimester nuchal translucency. However, the high proportion of false positives in this screening test involves that invasive sampling (amniocentesis or chorionic villi sampling to obtain a fetal karyotype) is often performed for normal fetuses. Furthermore, those invasive procedures are associated with a 0.2–0.5% risk of induced miscarriages [1]. Hence, researchers worldwide have long been seeking to design a more efficient test.

Since the discovery of the existence of circulating cell-free fetal DNA [2], several approaches have been used to deduce the number of fetal chromosomes on the basis of the DNA circulating in maternal plasma (despite the fact that the vast majority of DNA in the sample is maternal). To date, only molecule-counting based technologies relying on massively parallel sequencing (MPS) [3], or microarray-based method (MBM) [4] have yielded a useful screening tool. Even though MPS and MBM are able to address the problem from a technical point of view, it is unclear whether it will be applicable to large-scale (population-wide) screening of pregnant women: the technique is time-consuming, relatively costly and difficult to implement in genetic testing laboratories and is also hampered by ethical issues [5–8].

In digital PCR (dPCR), endpoint PCR is performed after the sample has been partitioned into thousands of individual reactors that (on average) contain a single molecule. It then becomes possible to evaluate the absolute quantification of the input DNA by counting the number of positive PCRs. Relative to conventional real-time PCR techniques, individual PCRs provide greater precision and resolution for detecting small concentration differences. The principle of dPCR was first described in 1999 [9]. However, the application of this technique to the field of non-invasive prenatal diagnosis of aneuploidies has been impaired by statistical issues and time-consuming practical issues. Given that cell free fetal DNA accounts for a weak proportion of the total cell-free DNA in maternal plasma, one needs to count thousands of molecules (i.e. positive PCRs) in order to reliability detect molecules originating from the trisomic chromosome. The lower the proportion of fetal DNA is, the higher the number of positive PCRs are required [10]. Lastly, given that the amounts of circulating cell-free fetal DNA in the plasma are very low, a large total number of PCRs have to be performed in order to achieve the requisite number of positive PCRs.

To date, the two main obstacles to the wider application of dPCR are (i) the technical difficulty of performing several thousands of PCRs, and (ii) the high number of positive PCRs required for statistical robustness. The first problem has been solved by recent technical breakthroughs in dPCR, such as droplet digital PCR (ddPCR), in which thousands of individual PCRs are performed in the same reaction well [11,12], which simplifies laboratory workflow. However, the number of positive PCRs is directly correlated with the number of available target DNA molecules in the sample, which is biologically limited. Hence, dPCR-based non-invasive prenatal testing (NIPT) for trisomy 21 has not previously been developed. Here, we designed a technical solution to this problem and tested it on plasma DNA samples from pregnant women, in order to establish whether our dPCR technique reliably discriminates between euploid samples and trisomic fetuses.

## Materials and Methods

### Patient recruitment

In this prospective study, we recruited pregnant women at high risk of chromosomal abnormalities and for whom invasive prenatal diagnosis was indicated on the basis of abnormal biochemical and/or ultrasound results. For all pregnancies, fetal karyotypes were available for final comparison with digital PCR results. The investigators performing the digital PCR experiments were not aware of the karyotype result. All patients gave their written, informed consent to participation in the study. Blood samples were collected (in EDTA tubes) either before the invasive prenatal diagnosis or at least two weeks afterwards. The study was approved by the local investigational review board (Comité de protection des personnes Ile de France XI) with the study approval reference 12079.

The median (range) age of gestation was 16 (9 to 37) weeks. Eighty eight samples (almost 42% of included samples) were obtained during the first trimester of pregnancy (until 14 weeks of gestation). Patients were addressed for high maternal serum markers (35% of cases), ultrasound abnormalities (45%), both anomalies (14%), familial history (3.5%) or other indications (3.5%).

### Sample processing and DNA extraction

One or two 4 ml EDTA tubes were collected for each patient, yielding an average of around 3 ml of plasma per patient. Blood samples were processed within two hours of collection. A three-step centrifugation protocol was set up for plasma recovery, with centrifugation (i) at 800 g for 10 min, (ii) at 1600 g for 10 min and (iii) at 16000 g for 10 min. Circulating DNA was extracted using the QIAamp^TM^ Circulating Nucleic Acid Kit (QIAGEN, Valencia, CA, USA), according to the manufacturer's instructions and resuspended in a final volume of 100 μl. The extracted DNA was stored below -20°C until processing.

### Droplet digital PCR

By generating uniform water-in-oil droplets, ddPCR partitions the target sequences into approximately 15,000 individual “nanoreactors” in the same well. Following an endpoint PCR using hydrolysis probes, each droplet is read and counted as positive or negative, depending on the presence or absence of target DNA sequence. It takes about an hour to prepare the droplets (including mix preparation), two hours for endpoint PCR and less than two minutes per reaction well for fluorescence reading. Absolute quantification is based on the number of positive droplets and Poisson sampling statistics, as follows: λ = -ln(1-k/n) where k is the number of positive droplets, n the total number of droplets and λ the mean copies per droplet.

We used the QX100 Droplet Digital PCR system (Bio-Rad Laboratories, Hercules, CA, USA). We designed a two-color octoplex PCR experiment that overcomes limitations on the amount of DNA by increasing the number of targets and thus increasing the number of positive PCRs. We used a set of four FAM TaqMan® hydrolysis assays for the *BRWD1*, *LTN1*, *NCAM2* and *RUNX1* genes (Assays identities respectively: Hs03026207_cn, Hs02872951_cn, Hs05556211_cn, Hs05550012_cn; Life Technologies, Carlsbad, CA, US) to detect chromosome 21. In order to open the field to the screening of both trisomy 21 and 18 at the same time, considering that the trisomy 18 is the second most frequent trisomy sometimes not identified with ultrasound during the 1st or 2nd trimester screening without increasing the final assay price, we designed a set of four VIC TaqMan® hydrolysis assays for the *CTIF*, *RIT2*, *SMAD4* and *TCF4* genes (Assays identities respectively: Hs06500717_cn, Hs06453395_cn, Hs06447834_cn, Hs00372815_cn; Life Technologies) to detect chromosome 18, which served as a reference chromosome. The chromosome ratio was calculated by dividing the amount of chromosome 21 by that of the reference chromosome.

However, to demonstrate that concept works whatever the chromosome chosen as a reference, we also validated our multiplex PCR system using chromosome 1 instead of chromosome 18 as the reference chromosome (S3 Table).

We first tested our multiplex design on serial dilutions of genomic DNA and artificial mixtures of trisomic and euploid DNA. Next, we established whether the multiplex assay was able to reliably discriminate between euploid and trisomic fetuses in plasma samples from pregnant women at high risk of chromosomal anomalies (according to biochemical and/or ultrasound screening) for whom a fetal karyotype was available.

### Validation of the multiplex PCR

We performed ddPCR on a series of dilutions of genomic DNA from peripheral lymphocytes using one, two, three or four TaqMan® assays per chromosome. In a final 20 μL reaction volume, we mixed 10 μL of 2X ddPCR Supermix for probes (Bio-Rad Laboratories, Inc.) with 0.5 μL of each TaqMan® assay (the final primers concentration was 450 nM and the final probes concentration was 125 nM), 2 μL of DNA and water up to 20 μL. The serial dilutions contained 2, 1, 0.5 and 0.25 ng/μL of DNA. Next, we tested the multiplex PCR assay's sensitivity for detecting small variations in the chromosome ratio in a very-low-concentration sample using artificial mixtures of trisomy 21 DNA and euploid DNA. We generated 0.5 ng/μL DNA mixtures containing 50%, 25%, 10%, 5% or 0% of trisomy 21 DNA. The 0% trisomy 21 sample was generated using a mix of 10% of one preparation of normal DNA and 90% of another preparation of normal DNA.

### ddPCR with circulating cell-free DNA

We composed a 20 μL reaction mix using 10 μL of 2X ddPCR Supermix for probes, 4 μL of an equimolar mix of the eight TaqMan® assays and 6 μL of plasma DNA. Next, we generated water-in-oil droplets using a commercial droplet generator (Bio-Rad Laboratories, Inc.). Eight replicates were performed for each sample. The PCR conditions were as follows: 95°C for 10 min, 40 cycles of 94°C for 30 sec and 60°C for 1 min, and then final extension at 98°C for 10 min. The fluorescence signal was measured and analyzed using a QX100 droplet reader and QuantaSoft software (both from Bio-Rad Laboratories, Inc.). A second run of 2 to 8 replicates was performed for samples with less than 5900 positive PCRs for the reference chromosome. Lastly, the chromosome ratio (based on Poisson statistics) was computed using the results of both runs. In this proof of concept study, we validated the PCR system only on samples from pregnancies with trisomy 21 or normal fetal karyotype.

### Data analysis

#### Validation of the multiplex PCR

Slopes for the concentrations (according to the dilution) were computed as a function of the number of probes used in the tested probe sets, and simultaneous confidence intervals for the slope ratios were computed and compared with those of the reference slope obtained with one probe for each chromosome [13].

For artificial mixtures of trisomic and euploid DNA, we performed local polynomial smoothing of the plot for observed and theoretical chromosome ratios with 95% confidence intervals, and then compared the observed curve with the expected curve.

#### ddPCR using circulating cell-free DNA

Chromosome ratios obtained from plasma DNA in euploid and trisomic groups were compared in a Wilcoxon rank-sum test. A ROC curve was then plotted.

## Results

### Validation of the multiplex PCR

#### Linearity regarding the number of probes in the reaction mix

**Fig 1:**
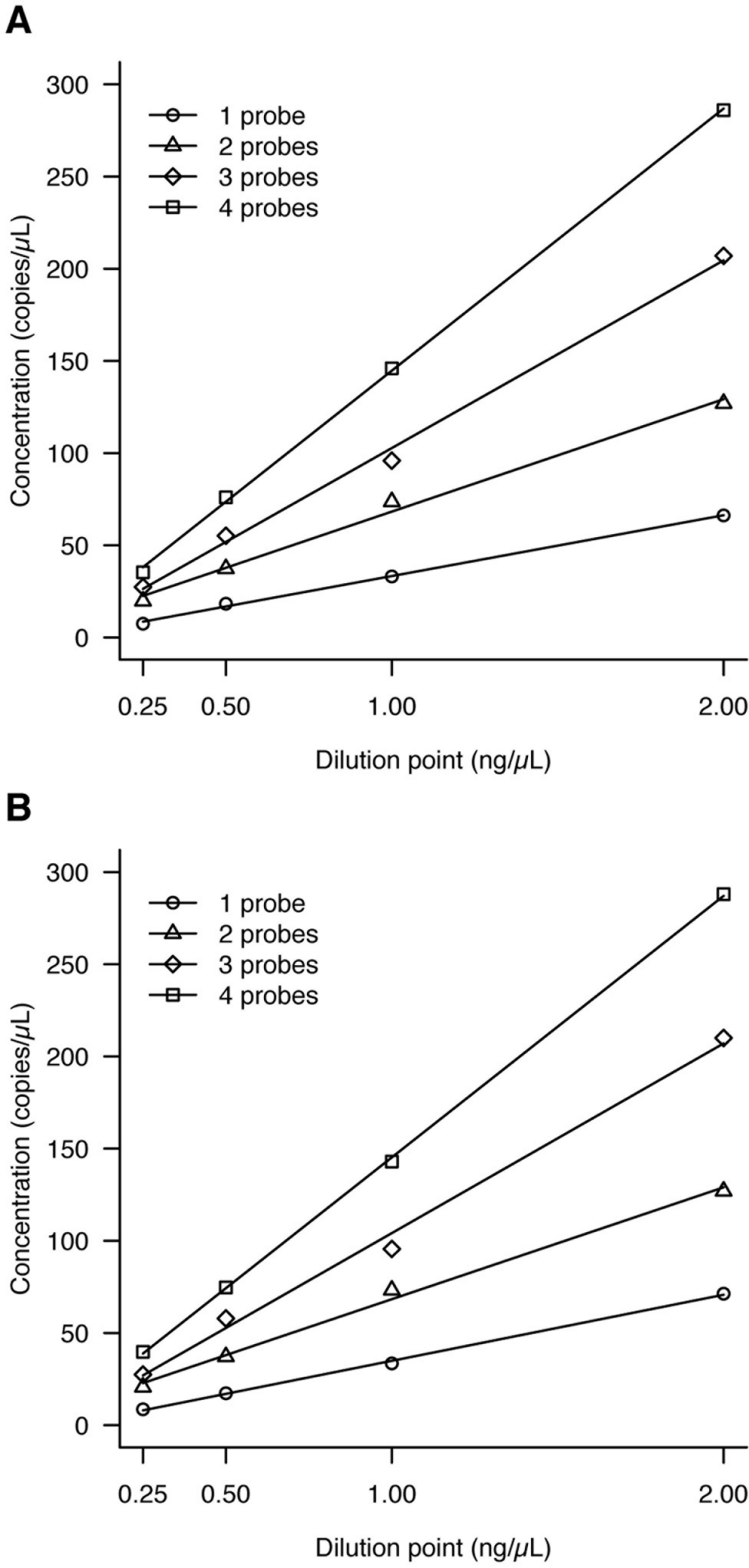
Validation of the multiplex PCR–serial dilutions for each set of probes. A: FAM probes (chromosome 21), B: VIC probes (reference chromosome).

We used series dilutions of genomic DNA (2.0, 1.0, 0.5 and 0.25 ng/μL) to validate the correlation between the expected and observed DNA concentrations for the different probe sets used in the reaction mix. For each set of n probes, we plotted the concentration curve of the series dilution (Fig 1) and calculated the slope. Then, we normalized the slopes results by dividing the slopes obtained with 2, 3 and 4 FAM or VIC probes targeting the same chromosome by the slope obtained with a single probe (FAM or VIC respectively) (Table 1). By this normalization, the slopes ratio should be equal to the number of probes used to target the same chromosome and this was the case with our results (Table 1). The slopes ratio when using two or three FAM probes for instance was respectively 2.05 and 3.22. Moreover, for a given fluorophore, positive droplets remained clearly distinguishable from negative droplets even when multiplexing the eight probes (see S1 Fig). Taken as a whole, these results demonstrate that our probes did not interfere with each other.

**Table 1:** Validation of the multiplex PCR.

	Slope (95%CI)	R^2^	Ratio of slopes (95%CI)
**FAM**			
1 probe	31.3 (26.7 to 35.8)	0.998	-
2 probes	64.2 (59.6 to 68.7)	0.994	2.05 (1.77 to 2.43)
3 probes	100.5 (96.0 to 105.0)	0.997	3.22 (2.78 to 3.80)
4 probes	142.0 (137.5 to 146.5)	>0.999	4.54 (3.93 to 5.38)
**VIC**			
1 probe	33.2 (28.2 to 38.2)	0.999	-
2 probes	64.1 (59.0 to 69.1)	0.995	1.93 (1.65 to 2.31)
3 probes	101.8 (96.7 to 106.8)	0.994	3.07 (2.64 to 3.68)
4 probes	142.2 (137.2 to 147.2)	>0.999	4.29 (3.69 to 5.13)

Ratio of slopes of the concentrations obtained for the multiplex assays in comparison to the simplex assay (the reference), and simultaneous confidence intervals.

#### Linearity regarding the percentage of trisomy 21

We next sought to assess the extent to which this PCR system was able to detect the small increment in chromosome ratio in trisomy 21 situations in samples mimicking plasma DNA from pregnant women. Given that this DNA is characterized by a low concentration of fetal DNA and a mix of maternal and fetal DNA, we created a model of mosaic trisomy by mixing DNA from euploid lymphocytes with DNA from trisomy 21 lymphocytes at very low concentrations and variable proportions of trisomy 21. After performing the ddPCR with our validated octoplex design, we compared the obtained chromosome ratios with the expected ratios. The theoretical ratio, depending on the fetal fraction, was calculated as follows:

r21/ref = [(n21fetal x p) + (n21maternal x (1-p))] / [(nreffetal x p) + (nrefmaternal x (1-p))], where p is the fetal fraction, n21 is the number of chromosomes 21 from maternal (n21maternal) or fetal (n21fetal) origin, and nref is the number of reference chromosome per diploid genome from maternal or fetal origin. The total number of molecules from a chromosome of interest being the addition of those from the fetus at a proportion of (p) and those from the mother at a proportion of (1-p).

In case of an euploid fetus, the theoretical ratio r21/ref is 1. In case of a trisomic fetus for chromosome 21 and a 10% fetal fraction (p = 0.1), the theoretical ratio can be calculated as r21/ref = 1 + p/2. = 1.05.

**Fig 2:**
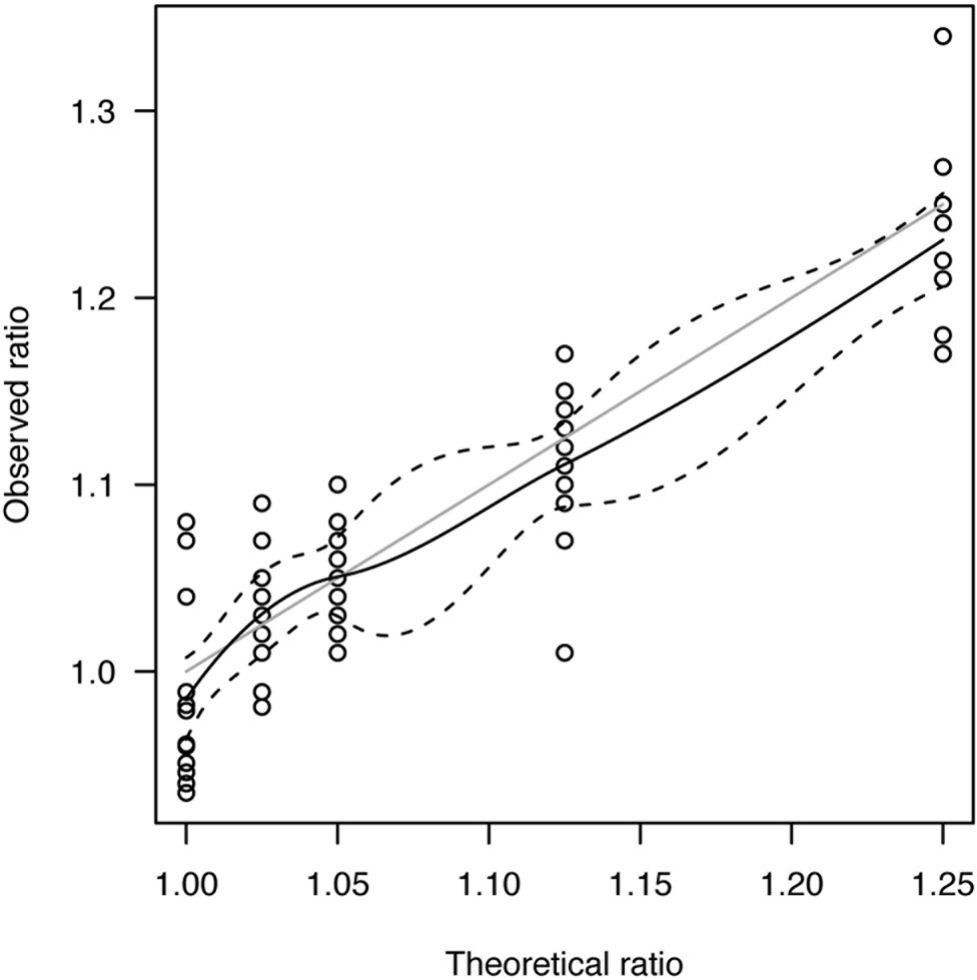
Observed vs. theoretical chromosome ratios for mixtures of 0.5 ng/μL DNA with a trisomy 21 DNA content of 0%, 5%, 10%, 25% and 50%. The solid black line corresponds to local polynomial smoothing of the curve with 95% confidence intervals (dashed lines), and the solid gray line represents the expected relationship.

We tested our multiplex assay with artificial mixtures of 0.5 ng/μL DNA (containing 50%, 25%, 10%, 5% or 0% of trisomy 21 DNA) and analyzed the chromosome ratios obtained for different replicates of the same point. Then, we analyzed the correlation between the observed ratios and the theoretical ones. When the correlation data were fitted to a linear model (black solid curve with the confidence interval in black dashed curves, in Fig 2), the estimated intercept and slope [95%CI] were respectively 0.06 [-0.07 to 0.19] and 0.94 [0.82 to 1.05] for theoretical values of 0 and 1 (solid gray curve in Fig 2), indicating that experimental ratios are not significantly different from theoretical ratios Similar results were obtained using mixtures of artificially degraded DNA samples (S2 Fig and S1 Table). Furthermore, these results show that the experimental ratios are similar to the theoretical ratios, but nevertheless not the same.

#### Validation with clinical samples

After validating the multiplex ddPCR design with genomic DNA samples, we tested it with plasma DNA samples from 213 pregnant women at high risk of chromosomal anomalies (according to biochemical and/or ultrasound screening). The fetal karyotype was normal in 192 cases and revealed trisomy 21 in 21 cases. For each sample, the pregnancy term at blood draw, the fetal karyotype and the results of ddPCR (according to the Minimum Information for Publication of Quantitative Digital PCR Experiments guidelines [14]) are shown in S2 Table.

Eight to sixteen replicates were performed for each sample. Approximately 10,000 to 18,000 water-in-oil droplets were generated per replicate, with an average of around 122,000 total droplets per sample. The median (range) total number of positive PCRs obtained for the reference chromosome was 13583 (1424 to 85214). We estimated the number of positive PCRs that had to be obtained to detect a small increase in the number of chromosome 21 molecules (compared with reference chromosome molecules) in cases of trisomy 21 with a low fetal fraction (10% and 5%). k_21_ and k_ref_ are the number of positive droplets for chromosome 21 and the reference chromosome, respectively, and n is the total number of PCRs (with about 256,000 PCRs for samples with sixteen replicates, which is the maximum number here). Although k_21_ and k_ref_ follow a binomial distribution, they can be considered as having a Gaussian distribution in the present context. For a single-sided test and with a 97.5% confidence interval, n21 is higher than nref when k_21_ > k_ref_ + 1.96*sigma_ref (where sigma_ref = √ (n*k_ref_/n*(1-k_ref_/n)). This condition is met when k_ref_ is 1600 (for a fetal fraction of 10%) or 5900 (for a fetal fraction of 5%).

**Fig 3:**
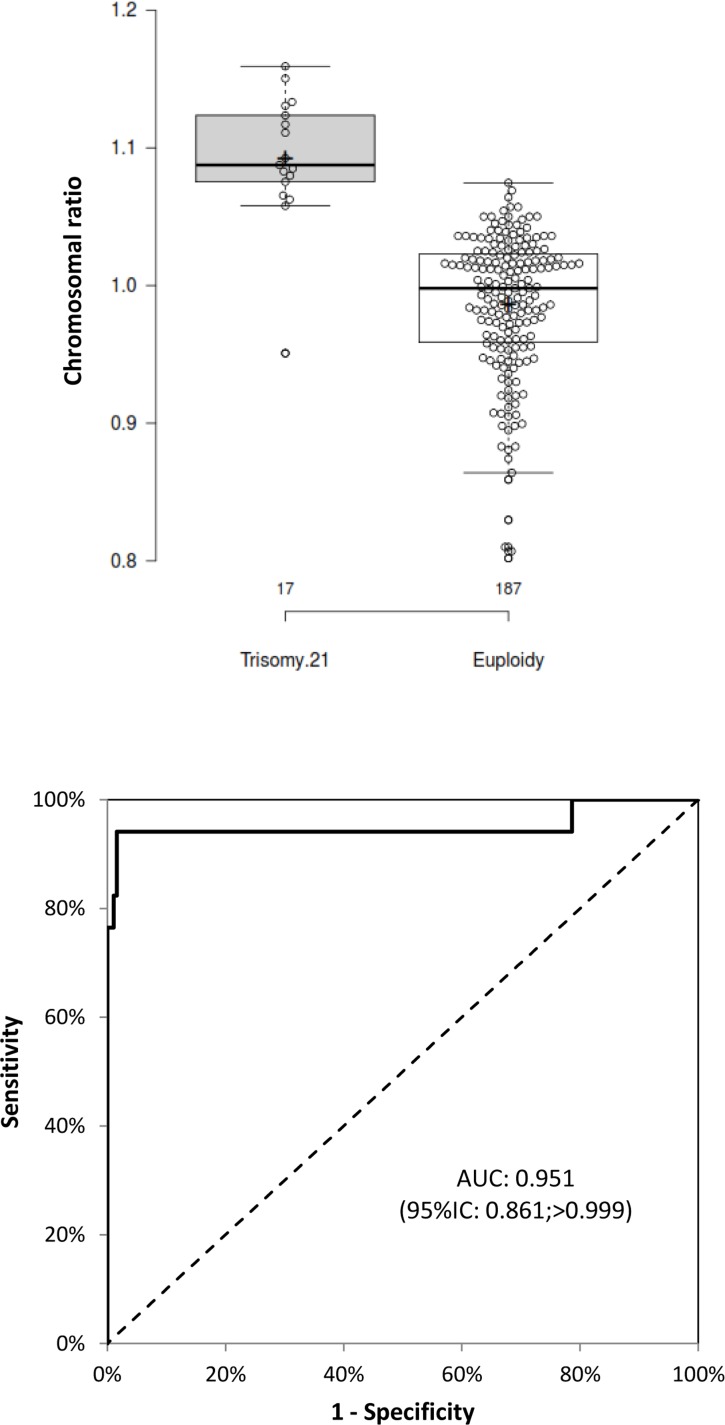
Box-and-whisker/Beeswarm plots of the distribution of chromosome ratios in the two groups (upper plot), and the ROC curve for the chromosome ratio as a predictor of trisomy 21 (lower plot) for samples exceeding the threshold for the number of positive PCRs. In the upper plot, the box displays the median [25^th^-75^th^ percentiles] for the distribution; the whiskers indicate the data points no further than 1.5 times the interquartile range from the box; crosses represent the group means; data points are plotted as open circles; n = 17 and 187 sample points for the trisomy 21 and normal group respectively. In the lower plot, the AUROC is given with its 95% confidence interval(AUC: Area Under the Curve).

For nine of the samples in our series (samples 1, 9, 40, 64, 66, 67, 76, 84 and 86)the number of positive PCRs was below 5900 (despite a second run). These quality control failures were excluded from further analysis (Fig 3).

The distribution of the chromosome ratio in trisomy 21 group was significantly shifted toward higher values, when compared with the euploid group (*p* << 0.0001) (Fig 3). A receiver operating characteristic (ROC) curve of the chromosome ratio was plotted as a predictor of trisomy 21 (Fig 3). The area under the ROC curve (AUROC: 0.951 (CI95%: 0.861;>0.999)) and the shape of the curve are characteristic of a very good screening test. Similar results were obtained when considering only 1^st^ trimester samples with more than 5900 positive PCRs (n = 85, *p* << 0.0001); AUROC: 0.920 (95%IC: 0.763;>0.999) (S3 Fig).

According to the ROC curve analysis, the best threshold for distinguishing between Trisomy 21 group and euploidy group is 1.057 (98% of specificity and 94% of sensitivity). With this threshold, the ratio of sample 41 is a false negative (fetal karyotype was 47,XY,+21). We sought to quantify the fetal fraction using a duplex hydrolysis probe assays targeting *SRY* (FAM) and *RNaseP* (VIC). None of the approximately 30,000 droplets was SRY-positive, demonstrating an undetectable fetal fraction. On the other hand, three results can be considered as false positive (samples 12, 51 and 145).

Among the nine samples excluded for insufficient number of positive PCRs, four were trisomic 21 (samples 1, 66, 67 and 76). Except for sample 66 with the lowest number of positive PCRs (~2800 far from the 5900 required for distinguishing between trisomic and euploid situations), for three other samples the chromosomal ratio was in favor of a trisomy 21.

Using chromosome 1 as the reference chromosome we tested some plasma DNA samples (n = 20 among which 3 trisomy 21). Ratio for trisomy 21 (n = 3) and euploid fetus (n = 17) were respectively at 1.10 and 1.02 (p = 0.007) (S3 Table)

## Discussion

In the present study, we developed a novel ddPCR-based method for the non-invasive prenatal screening of trisomy 21 using DNA from maternal plasma.

Digital PCR has already been proposed as an alternative technique for aneuploidy NIPT. Fan et al. showed that this approach is precise enough to detect small variation in the chromosome ratio caused by trisomy [15, 16]. Firstly, calculations showed that thousands of positive partitions (the droplets, in ddPCR) were needed to achieve statistical significance with high confidence, meaning that thousands of PCRs need to be processed [15, 16]. Secondly, and despite great technical progress in the field of dPCR (enabling thousands of PCRs to be performed in the same well), the number of available target DNAs is sometimes insufficient. By targeting several genes on the same chromosome with a multiplex of probes labeled with the same fluorophore, we overcame this limitation by substantially increasing the number of positive droplets for a given amount of input DNA. It is important to note that the number of positive droplets was correlated with the number of probes, thus indicating an absence of interference. The choice of primers and probes is also crucial; these must target small sequences, since the average length of fetal DNA is about 140 bp [3, 17]. Hence, the amplicons used in the present study did not exceed 75 bp in length. Furthermore, the target sequences are located outside of regions affected by known, benign copy number variations. Lastly, it is essential to avoid interference by targeting genes that lack homology. Our PCR optimization experiments showed that a combination of multiplexing and automated partitioning of template DNA can be used for NIPT for trisomy 21.

In our validation study, we demonstrated that the multiplexed ddPCR approach provides statistically significant discrimination between trisomic and euploid samples. The only false negative sample came from a pregnant woman bearing a trisomic male fetus; consistently, the ddPCR assay for SRY/RNaseP could not detect fetal DNA. This observation argues for systematic estimation of the fetal fraction as a pre-analytical quality check. Several researchers have shown that this can be achieved by targeting a differentially methylated region within the *RASSF1A* gene's promoter; the sequence is hypermethylated in placenta lymphocytes and hypomethylated in maternal lymphocytes. Chan et al. made use of this methylation pattern to perform a methylation-sensitive restriction enzyme digestion and then detect placenta-derived, hypermethylated *RASSF1A* sequences in maternal plasma. They demonstrated that this universal marker is useful for the detection of false-negative results caused by low fetal DNA concentrations in maternal plasma [18]. We are now developing this step in order to further increase the reliability of our ddPCR assay. Given that the threshold to be used in clinical applications will depend on the fetal fraction, we are evaluating whether the estimation of the fetal fraction, using a quantification based on the differential methylation of RASSF1A promoter between maternal and fetal DNA, can be combined to the chromosomal ratio in order to further strengthen the test.

Although we could demonstrate in this preliminary pilot study that it was possible to obtain a significantly different chromosome 21/reference chromosome ratio between a group of euploid and trisomic samples after stratification according to the karyotype result (Fig 3), a larger cohort will obviously be paramount in order to determine an optimal threshold (for maximizing sensitivity and minimizing the false positive rate) for prospectively classify the samples. However, our ROC curve demonstrates that our assay already has the characteristics of a very good screening test—far better than the current triple-test strategy. From a healthcare point of view, an improvement in screening efficiency only makes sense if it can be made available to all pregnant women. A screening strategy based on this ddPCR assay would both increase the number of screened, abnormal fetuses and decrease the number of invasive procedures (and thus induced miscarriages). In contrast, if NIPT is restricted to at-risk women (because of the high cost of MPS-based technologies), the rate of fetal loss would fall but the screening performance would not improve. In this respect, the ddPCR assay's cost per sample (between 30 and 40 euros, depending on the number of PCR reactions required to obtain 5900 positive droplets) and workflow match the requirements of a population-wide screening test. The workflow is straightforward because a result can be obtained within a day. Up to 12 samples can be run at once. The test's efficiency and throughput could be increased further by automating the reaction mix preparation and droplet generation. The data analysis is very simple and does not require intensive computation or large storage facilities. The equipment is also relatively affordable, and easy to install and implement in routine laboratory practice. Lastly, from an ethical point of view, this test is compatible with the large-scale screening of a low-risk population of pregnant women because there is no longer any need to access sequence information.

Because it has been demonstrated that all the fetal genome is entirely represented among the circulating DNA [19], the choice of the reference chromosome should not be an issue. Hence we selected for this proof of concept study the chromosome 18 as reference chromosome in order to open the field to the screening of both trisomy 21 and 18 at the same time. We are aware of concerns regarding the frequency of trisomy 18 in confined placental mosaicism [20–22], but this event can result in a false positive for trisomy 18 not for trisomy 21 which is studied here. A false negative situation for trisomy 21 due to confined placental trisomy 18 is *a priori* not possible since these two conditions have never been observed concomitantly. However, we validated our multiplex PCR system using chromosome 1 as the reference chromosome and demonstrate that this system is valid whatever the chromosome tested but the ratio threshold will probably differ according to the probes used.

This proof of concept study demonstrates that multiplex ddPCR is a promising approach for NIPT for trisomy 21, which can be extended to the detection of other aneuploidies and large copy number variations. Because MPS or MBM have already proven their efficiency in this purpose, practical use of ddPCR in clinical setting will probably depend upon technical optimization of the technology and on pricing evolution of MPS or MBM to make it (or not) available to all pregnant women. Furthermore, further experiments will be necessary for trisomy 18 screening.

## Supporting Information

S1 Fig A two-dimensional representation of the fluorescence intensity obtained for plasma DNA using the octoplex ddPCR assay. The y and x axes correspond to the FAM and VIC intensities, respectively. Negative droplets (grey dots) and positive ones (blue dots for FAM+ only, green dots for VIC+ only and brown for FAM+ and VIC+) are assigned as a function of the FAM and VIC florescence amplitudes.
(TIF)

S2 Fig Electrophoresis analysis, by the Agilent 2100 Bioanalyzer system, of an artificially degraded genomic DNA using the Covaris ultrasonicator, in order to mimic the plasma DNA degradation profile. (TIF)

S3 Fig Box and whisker plots of the distribution of chromosome ratios in the two groups (upper plot) and the ROC curve for the chromosome ratio as a predictor of trisomy 21 (lower plot) for first trimester pregnancies samples. In the upper plot, the box displays the median [25^th^-75^th^ percentiles] for the distribution; the whiskers indicate the data points no further than 1.5 times the interquartile range from the box; crosses represent the group means; data points are plotted as open circles (n = 10) and sample points (n = 75) for the trisomy 21 and normal group respectively. In the lower plot, the AUROC is given with its 95% confidence interval (AUC: Area Under the Curve).
(TIF)

S1 Table Results of chromosomal ratios for mixtures of 0.5 ng/μL artificially degraded DNA with a trisomy 21 DNA content of 0%, 5%, 10%, 25% and 50%. (DOCX)

S2 Table Validation study: the results of ddPCR on plasma DNA. k 21 and k ref are the number of positive droplets for chromosome 21 and the reference chromosome, respectively. n is the total number of PCRs. λ 21 and λ ref are the estimated mean number of copies per droplet, according to the Poisson distribution. NA = not available.
(DOCX)

S3 Table Results of ddPCR on a set of plasma DNA using chromosome 1 as a reference chromosome. We used a set of three FAM TaqMan® hydrolysis assays for the *APP*, *BRWD1* and *RUNX1* genes (Assays identities respectively: Hs01344980_cn, Hs03026207_cn and Hs05550012_cn; Life Technologies, Carlsbad, CA, US) and three VIC TaqMan® hydrolysis assays for the *ASTN1*, *FAF1* and *PUM1* genes (Assays identities respectively: Hs05795637_cn, Hs06521574_cn and Hs06604919_cn; Life Technologies, Carlsbad, CA, US). ddPCR protocol was the same as described in the Materials and Methods section. k 21 and k ref are the number of positive droplets for chromosome 21 and the reference chromosome, respectively. n is the total number of PCRs. λ 21 and λ ref are the estimated mean number of copies per droplet, according to the Poisson distribution.
(DOCX)
