# Supplementary figures and images for: Could Digital PCR Be an Alternative as a Non-Invasive Prenatal Test for Trisomy 21: A Proof of Concept Study
Source: PLoS One. 2016 May 11;11(5):e0155009. doi: 10.1371/journal.pone.0155009 (PMC4864235; doi:10.1371/journal.pone.0155009)

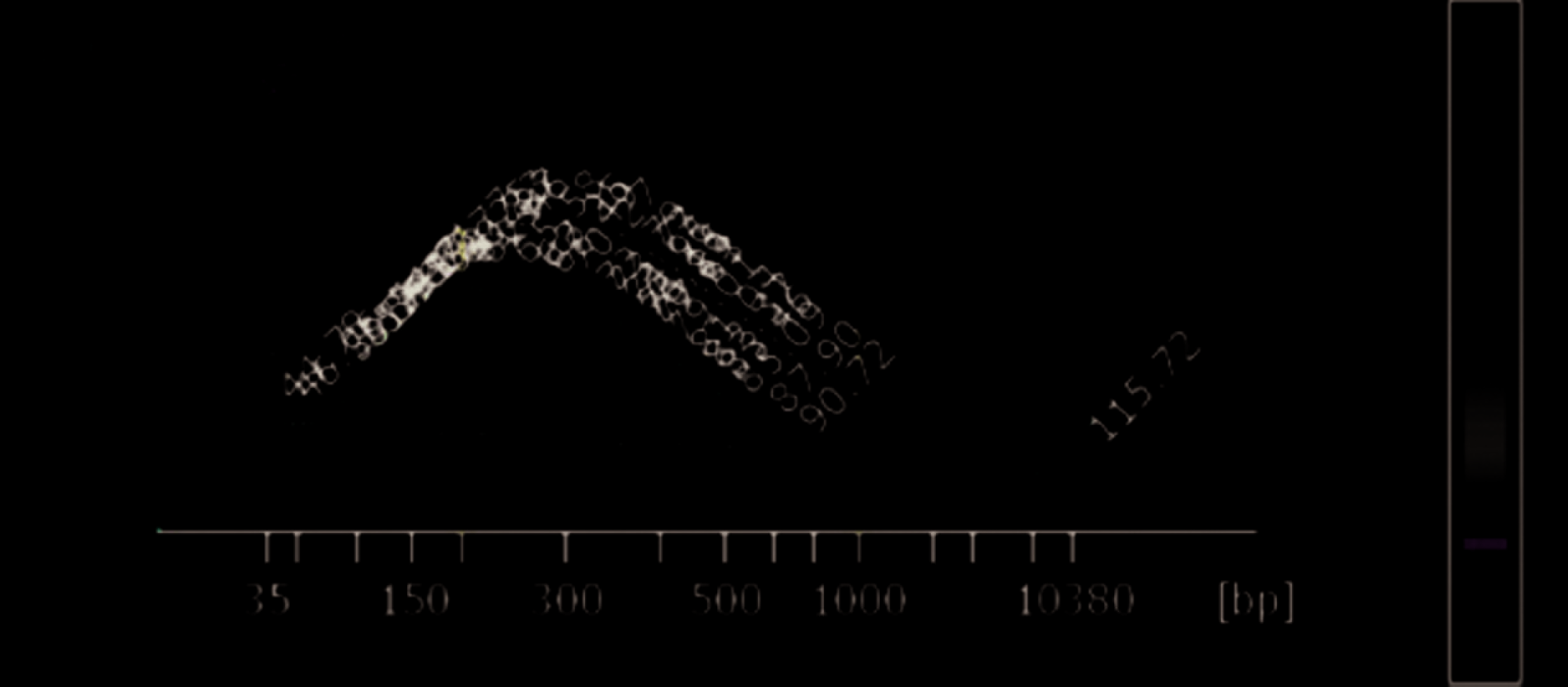

Supplement: S2 Fig — (TIF) [file pone.0155009.s002.tif]
