# Supplementary material for: Could Digital PCR Be an Alternative as a Non-Invasive Prenatal Test for Trisomy 21: A Proof of Concept Study
Source: PLoS One. 2016 May 11;11(5):e0155009. doi: 10.1371/journal.pone.0155009 (PMC4864235; doi:10.1371/journal.pone.0155009)
Supplement: S1 Table — (DOCX) [file pone.0155009.s004.docx]

**Supplementary Table 1.** Results of chromosomal ratios for mixtures of 0.5 ng/µL artificially degraded DNA with a trisomy 21 DNA content of 0%, 5%, 10%, 25% and 50%.

| **% of Trisomy 21** | **chr 21/ref theoritical ratio** | **chr 21/ref experimental ratio** | **SD** | **CI95 min** | **CI95 max** | **N** |
| --- | --- | --- | --- | --- | --- | --- |
| **0%** | 1 | 1.022 | 0.020 | 0.982 | 1.062 | 6 |
| **5%** | 1.025 | 1.038 | 0.038 | 0.962 | 1.113 | 8 |
| **10%** | 1.05 | 1.060 | 0.024 | 1.014 | 1.106 | 5 |
| **25%** | 1.125 | 1.133 | 0.039 | 1.057 | 1.208 | 4 |
| **50%** | 1.25 | 1.230 | 0.020 | 1.191 | 1.269 | 3 |
