# Supplementary material for: Could Digital PCR Be an Alternative as a Non-Invasive Prenatal Test for Trisomy 21: A Proof of Concept Study
Source: PLoS One. 2016 May 11;11(5):e0155009. doi: 10.1371/journal.pone.0155009 (PMC4864235; doi:10.1371/journal.pone.0155009)
Supplement: S2 Table — k 21 and k ref are the number of positive droplets for chromosome 21 and the reference chromosome, respectively. n is the total number of PCRs. λ 21 and λ ref are the estimated mean number of copies per droplet, according to the Poisson distribution. NA = not available. (DOCX) [file pone.0155009.s005.docx]

**Supplementary Table 2: Validation study – results of ddPCR on plasma DNA**

|  | | | | | |  |  |  |  |
| --- | --- | --- | --- | --- | --- | --- | --- | --- | --- |
| **Sample** | **Fetal ploïdy** | **Gestational age (GW)** | **k 21** | **k ref** | **n** | **λ 21** | **λ ref** | **Chromosomal ratio (21/ref)** | **Number of replicates** |
| **1** | T21 | 23 | 4239 | 3976 | 103441 | 0.0418 | 0.0392 | 1.0675 | 8 |
| **2** | T21 | 11 | 8685 | 8038 | 153237 | 0.0583 | 0.0539 | 1.0829 | 10 |
| **3** | N | 23 | 7121 | 6997 | 176767 | 0.0411 | 0.0404 | 1.0181 | 12 |
| **4** | N | 20 | 14717 | 14182 | 127274 | 0.1229 | 0.1181 | 1.0401 | 8 |
| **5** | T21 | NA | 12771 | 12029 | 116485 | 0.1161 | 0.1090 | 1.0654 | 8 |
| **6** | T21 | NA | 6750 | 6358 | 255026 | 0.0268 | 0.0252 | 1.0625 | 19 |
| **7** | N | 9 | 8093 | 7796 | 208381 | 0.0396 | 0.0381 | 1.0389 | 14 |
| **8** | N | 15 | 7713 | 7626 | 179928 | 0.0438 | 0.0433 | 1.0117 | 12 |
| **9** | N | 16 | 5517 | 5437 | 241498 | 0.0231 | 0.0228 | 1.0149 | 16 |
| **10** | T21 | 11 | 9913 | 9247 | 116398 | 0.0890 | 0.0828 | 1.0753 | 8 |
| **11** | N | 14 | 7426 | 7479 | 152706 | 0.0499 | 0.0502 | 0.9927 | 10 |
| **12** | N | 20 | 30389 | 28580 | 110899 | 0.3202 | 0.2980 | 1.0746 | 8 |
| **13** | N | 25 | 7564 | 7319 | 119277 | 0.0655 | 0.0633 | 1.0346 | 8 |
| **14** | N | 23 | 8167 | 8055 | 177894 | 0.0470 | 0.0463 | 1.0142 | 12 |
| **15** | T21 | 10 | 7621 | 6807 | 120602 | 0.0653 | 0.0581 | 1.1236 | 8 |
| **16** | N | 23 | 9861 | 9721 | 161690 | 0.0629 | 0.0620 | 1.0149 | 12 |
| **17** | T21 | 11 | 6875 | 6378 | 152764 | 0.0460 | 0.0426 | 1.0797 | 10 |
| **18** | N | 24 | 12916 | 12331 | 124844 | 0.1092 | 0.1040 | 1.0501 | 8 |
| **19** | N | 12 | 7379 | 7476 | 203455 | 0.0369 | 0.0374 | 0.9868 | 14 |
| **20** | N | 23 | 12401 | 11794 | 124406 | 0.1050 | 0.0996 | 1.0543 | 8 |
| **21** | N | 11 | 7931 | 8226 | 175670 | 0.0462 | 0.0480 | 0.9633 | 12 |
| **22** | N | 17 | 7320 | 7543 | 167414 | 0.0447 | 0.0461 | 0.9698 | 12 |
| **23** | N | 11 | 7533 | 7285 | 117520 | 0.0662 | 0.0640 | 1.0352 | 8 |
| **24** | N | 10 | 9311 | 9050 | 115845 | 0.0838 | 0.0813 | 1.0301 | 8 |
| **25** | N | 11 | 7162 | 7048 | 117833 | 0.0627 | 0.0617 | 1.0167 | 8 |
| **26** | N | 9 | 8230 | 7979 | 117849 | 0.0724 | 0.0701 | 1.0326 | 8 |
| **27** | N | 15 | 12441 | 12113 | 104603 | 0.1266 | 0.1231 | 1.0289 | 7 |
| **28** | N | 16 | 8135 | 8093 | 137396 | 0.0610 | 0.0607 | 1.0054 | 9 |
| **29** | N | 16 | 11161 | 11602 | 123267 | 0.0949 | 0.0988 | 0.9601 | 8 |
| **30** | N | NA | 40813 | 44129 | 117247 | 0.4279 | 0.4722 | 0.9061 | 8 |
| **31** | N | 16 | 30693 | 33740 | 162617 | 0.2092 | 0.2325 | 0.8995 | 12 |
| **32** | N | 21 | 11058 | 12588 | 167056 | 0.0685 | 0.0783 | 0.8742 | 12 |
| **33** | N | 13 | 7228 | 7331 | 177775 | 0.0415 | 0.0421 | 0.9857 | 14 |
| **34** | N | 17 | 8160 | 8600 | 156669 | 0.0535 | 0.0565 | 0.9474 | 12 |
| **35** | N | 14 | 10317 | 11172 | 165251 | 0.0645 | 0.0700 | 0.9209 | 12 |
| **36** | N | 12 | 23749 | 25399 | 166175 | 0.1542 | 0.1659 | 0.9297 | 12 |
| **37** | N | 12 | 8772 | 9903 | 188121 | 0.0478 | 0.0541 | 0.8830 | 12 |
| **38** | N | 25 | 14844 | 17641 | 113168 | 0.1406 | 0.1695 | 0.8297 | 8 |
| **39** | N | 18 | 41498 | 44748 | 112006 | 0.4628 | 0.5100 | 0.9075 | 8 |
| **40** | N | 12 | 4489 | 4303 | 220383 | 0.0206 | 0.0197 | 1.0437 | 16 |
| **41** | T21 | 12 | 83159 | 85214 | 115297 | 1.2775 | 1.3436 | 0.9508 | 8 |
| **42** | N | 23 | 10519 | 11864 | 108199 | 0.1023 | 0.1161 | 0.8806 | 8 |
| **Sample** | **Fetal ploïdy** | **Gestational age (GW)** | **k 21** | **k ref** | **n** | **λ 21** | **λ ref** | **Chromosomal ratio (21/ref)** | **Number of replicates** |
| **43** | T21 | 13 | 6912 | 6125 | 221049 | 0.0318 | 0.0281 | 1.1306 | 16 |
| **44** | N | 27 | 19009 | 19809 | 118341 | 0.1751 | 0.1832 | 0.9559 | 8 |
| **45** | N | 37 | 12175 | 13518 | 113442 | 0.1135 | 0.1269 | 0.8948 | 8 |
| **46** | N | 26 | 10024 | 10628 | 108714 | 0.0967 | 0.1029 | 0.9403 | 8 |
| **47** | N | 23 | 8440 | 10320 | 106264 | 0.0828 | 0.1022 | 0.8101 | 8 |
| **48** | T21 | 18 | 7278 | 6355 | 108800 | 0.0692 | 0.0602 | 1.1504 | 8 |
| **49** | N | 21 | 7695 | 7688 | 128210 | 0.0619 | 0.0618 | 1.0009 | 9 |
| **50** | N | 30 | 16371 | 16115 | 109477 | 0.1620 | 0.1592 | 1.0172 | 8 |
| **51** | N | 22 | 7671 | 7225 | 110711 | 0.0718 | 0.0675 | 1.0640 | 8 |
| **52** | T21 | 19 | 7860 | 7210 | 156650 | 0.0515 | 0.0471 | 1.0925 | 12 |
| **53** | N | 17 | 16323 | 16109 | 97163 | 0.1839 | 0.1813 | 1.0146 | 8 |
| **54** | T21 | 12 | 10326 | 9292 | 109197 | 0.0993 | 0.0889 | 1.1170 | 8 |
| **55** | T21 | 11 | 7576 | 6560 | 141506 | 0.0550 | 0.0475 | 1.1592 | 10 |
| **56** | N | 23 | 8051 | 8341 | 123347 | 0.0675 | 0.0700 | 0.9640 | 8 |
| **57** | N | 21 | 13480 | 13130 | 120550 | 0.1186 | 0.1153 | 1.0283 | 8 |
| **58** | N | 31 | 28179 | 27921 | 114207 | 0.2833 | 0.2803 | 1.0107 | 8 |
| **59** | N | 12 | 6907 | 6821 | 114909 | 0.0620 | 0.0612 | 1.0130 | 8 |
| **60** | N | 21 | 7506 | 7541 | 113174 | 0.0686 | 0.0690 | 0.9952 | 8 |
| **61** | N | 17 | 8619 | 8333 | 229503 | 0.0383 | 0.0370 | 1.0350 | 16 |
| **62** | N | 12 | 7526 | 7197 | 170736 | 0.0451 | 0.0431 | 1.0468 | 12 |
| **63** | N | 21 | 10907 | 10701 | 117303 | 0.0976 | 0.0957 | 1.0202 | 8 |
| **64** | N | 19 | 2688 | 2561 | 244013 | 0.0111 | 0.0106 | 1.0499 | 16 |
| **65** | N | 10 | 10807 | 10373 | 110249 | 0.1032 | 0.0988 | 1.0441 | 8 |
| **66** | T21 | 14 | 2844 | 2817 | 116882 | 0.0246 | 0.0244 | 1.0097 | 8 |
| **67** | T21 | 10 | 4450 | 4088 | 224995 | 0.0200 | 0.0183 | 1.0894 | 16 |
| **68** | N | 20 | 7067 | 7562 | 115743 | 0.0630 | 0.0676 | 0.9324 | 8 |
| **69** | N | 20 | 10996 | 11192 | 230548 | 0.0489 | 0.0498 | 0.9821 | 16 |
| **70** | N | 14 | 7726 | 7477 | 135157 | 0.0589 | 0.0569 | 1.0343 | 9 |
| **71** | T21 | 11 | 7614 | 6737 | 165529 | 0.0471 | 0.0416 | 1.1333 | 11 |
| **72** | N | 16 | 39711 | 46668 | 96046 | 0.5335 | 0.6653 | 0.8019 | 8 |
| **73** | N | 30 | 15012 | 14652 | 111065 | 0.1452 | 0.1415 | 1.0264 | 8 |
| **74** | N | 11 | 8008 | 7977 | 143279 | 0.0575 | 0.0573 | 1.0040 | 10 |
| **75** | N | 14 | 10264 | 10332 | 117994 | 0.0910 | 0.0916 | 0.9931 | 8 |
| **76** | T21 | 17 | 5520 | 5149 | 126140 | 0.0447 | 0.0417 | 1.0737 | 8 |
| **77** | N | 14 | 9062 | 8954 | 178262 | 0.0522 | 0.0515 | 1.0124 | 12 |
| **78** | N | 16 | 40133 | 39780 | 104910 | 0.4822 | 0.4767 | 1.0114 | 8 |
| **79** | N | 14 | 22101 | 22300 | 114008 | 0.2155 | 0.2177 | 0.9900 | 8 |
| **80** | N | 14 | 7532 | 7837 | 138182 | 0.0560 | 0.0584 | 0.9600 | 10 |
| **81** | N | 14 | 13918 | 13823 | 111518 | 0.1333 | 0.1323 | 1.0074 | 8 |
| **82** | N | 21 | 7801 | 7438 | 166949 | 0.0479 | 0.0456 | 1.0500 | 12 |
| **83** | N | 11 | 8463 | 8187 | 163710 | 0.0531 | 0.0513 | 1.0346 | 12 |
| **84** | N | 24 | 1393 | 1424 | 90049 | 0.0156 | 0.0159 | 0.9781 | 7 |
| **85** | T21 | 24 | 18968 | 18024 | 106177 | 0.1968 | 0.1860 | 1.0579 | 8 |
| **86** | N | 22 | 4791 | 4956 | 106672 | 0.0460 | 0.0476 | 0.9659 | 8 |
| **Sample** | **Fetal ploïdy** | **Gestational age (GW)** | **k 21** | **k ref** | **n** | **λ 21** | **λ ref** | **Chromosomal ratio (21/ref)** | **Number of replicates** |
| **87** | N | 11 | 8648 | 8770 | 146527 | 0.0608 | 0.0617 | 0.9857 | 10 |
| **88** | N | 10 | 14723 | 14545 | 103559 | 0.1533 | 0.1513 | 1.0132 | 8 |
| **89** | N | 15 | 17675 | 17692 | 108244 | 0.1783 | 0.1785 | 0.9989 | 8 |
| **90** | N | 11 | 7360 | 7205 | 157117 | 0.0480 | 0.0469 | 1.0220 | 13 |
| **91** | N | 11 | 7088 | 6962 | 77743 | 0.0956 | 0.0938 | 1.0190 | 8 |
| **92** | N | 11 | 13169 | 14020 | 130430 | 0.1064 | 0.1137 | 0.9359 | 8 |
| **93** | N | 11 | 23081 | 24255 | 132973 | 0.1906 | 0.2014 | 0.9467 | 8 |
| **94** | N | 10 | 10254 | 11148 | 250609 | 0.0418 | 0.0455 | 0.9181 | 16 |
| **95** | N | 11 | 9474 | 9999 | 130872 | 0.0751 | 0.0795 | 0.9455 | 8 |
| **96** | N | 13 | 13508 | 13618 | 125148 | 0.1142 | 0.1152 | 0.9914 | 8 |
| **97** | N | 22 | 6686 | 6867 | 247922 | 0.0273 | 0.0281 | 0.9733 | 16 |
| **98** | N | NA | 7696 | 7563 | 173215 | 0.0454 | 0.0446 | 1.0180 | 12 |
| **99** | N | 16 | 11694 | 12763 | 116051 | 0.1062 | 0.1165 | 0.9116 | 8 |
| **100** | T21 | 16 | 7925 | 7301 | 168398 | 0.0482 | 0.0443 | 1.0876 | 12 |
| **101** | N | 13 | 28393 | 28665 | 108742 | 0.3026 | 0.3060 | 0.9889 | 8 |
| **102** | N | 11 | 7533 | 7136 | 133965 | 0.0579 | 0.0547 | 1.0570 | 8 |
| **103** | N | 13 | 17851 | 18896 | 130405 | 0.1472 | 0.1565 | 0.9400 | 8 |
| **104** | N | 14 | 20265 | 20743 | 134330 | 0.1635 | 0.1677 | 0.9750 | 8 |
| **105** | N | 25 | 12601 | 13131 | 131946 | 0.1004 | 0.1048 | 0.9580 | 8 |
| **106** | N | 13 | 11159 | 10892 | 108053 | 0.1090 | 0.1063 | 1.0260 | 8 |
| **107** | N | 31 | 15055 | 14794 | 101948 | 0.1598 | 0.1568 | 1.0190 | 8 |
| **108** | N | 16 | 19812 | 20170 | 119421 | 0.1814 | 0.1850 | 0.9810 | 8 |
| **109** | N | 25 | 10573 | 10958 | 138944 | 0.0791 | 0.0822 | 0.9630 | 8 |
| **110** | N | 13 | 11651 | 11971 | 132960 | 0.0917 | 0.0943 | 0.9720 | 8 |
| **111** | N | 12 | 10566 | 11035 | 107620 | 0.1033 | 0.1082 | 0.9550 | 6 |
| **112** | N | 12 | 7494 | 8165 | 97434 | 0.0800 | 0.0875 | 0.9140 | 6 |
| **113** | N | 23 | 11356 | 12281 | 95461 | 0.1267 | 0.1377 | 0.9200 | 6 |
| **114** | N | 12 | 16353 | 17224 | 111782 | 0.1582 | 0.1673 | 0.9450 | 6 |
| **115** | N | 13 | 10534 | 11019 | 108821 | 0.1018 | 0.1068 | 0.9540 | 6 |
| **116** | N | 18 | 8548 | 8774 | 103311 | 0.0864 | 0.0888 | 0.9730 | 6 |
| **117** | N | 13 | 10587 | 11126 | 105607 | 0.1056 | 0.1113 | 0.9490 | 6 |
| **118** | N | 25 | 12700 | 13401 | 98946 | 0.1374 | 0.1455 | 0.9440 | 6 |
| **119** | N | 30 | 6554 | 6907 | 83921 | 0.0813 | 0.0859 | 0.9470 | 6 |
| **120** | N | 13 | 12329 | 12536 | 94097 | 0.1404 | 0.1430 | 0.9820 | 6 |
| **121** | N | 13 | 9182 | 9723 | 100305 | 0.0960 | 0.1020 | 0.9420 | 6 |
| **122** | N | 16 | 5376 | 5926 | 113238 | 0.0486 | 0.0538 | 0.9050 | 6 |
| **123** | N | 19 | 9338 | 11442 | 106925 | 0.0914 | 0.1132 | 0.8070 | 6 |
| **124** | N | 25 | 15457 | 17330 | 111031 | 0.1499 | 0.1697 | 0.8830 | 6 |
| **125** | N | 16 | 7819 | 8188 | 95400 | 0.0855 | 0.0897 | 0.9530 | 6 |
| **126** | N | 26 | 5715 | 6280 | 97684 | 0.0603 | 0.0664 | 0.9070 | 6 |
| **127** | N | 15 | 9800 | 10234 | 96309 | 0.1073 | 0.1123 | 0.9550 | 6 |
| **128** | N | 13 | 8645 | 9935 | 97149 | 0.0932 | 0.1079 | 0.8640 | 6 |
| **129** | N | 16 | 15139 | 16183 | 101294 | 0.1619 | 0.1741 | 0.9300 | 6 |
| **130** | N | 11 | 6463 | 7170 | 100579 | 0.0664 | 0.0740 | 0.8980 | 6 |
| **Sample** | **Fetal ploïdy** | **Gestational age (GW)** | **k 21** | **k ref** | **n** | **λ 21** | **λ ref** | **Chromosomal ratio (21/ref)** | **Number of replicates** |
| **131** | N | 23 | 5836 | 6477 | 96950 | 0.0621 | 0.0691 | 0.8980 | 6 |
| **132** | N | 19 | 6889 | 6993 | 84619 | 0.0849 | 0.0863 | 0.9840 | 6 |
| **133** | N | 15 | 16500 | 17790 | 96918 | 0.1866 | 0.2028 | 0.9200 | 6 |
| **134** | N | 21 | 16744 | 19170 | 87157 | 0.2133 | 0.2484 | 0.8590 | 6 |
| **135** | N | 13 | 9161 | 9877 | 99800 | 0.0963 | 0.1042 | 0.9240 | 6 |
| **136** | N | 14 | 13984 | 14291 | 107359 | 0.1396 | 0.1428 | 0.9770 | 6 |
| **137** | N | 26 | 9194 | 9009 | 103510 | 0.0930 | 0.0911 | 1.0220 | 6 |
| **138** | N | 25 | 14219 | 14059 | 101118 | 0.1515 | 0.1497 | 1.0120 | 6 |
| **139** | N | 24 | 14469 | 14708 | 99913 | 0.1564 | 0.1592 | 0.9820 | 6 |
| **140** | N | 15 | 6879 | 6647 | 108754 | 0.0653 | 0.0631 | 1.0360 | 6 |
| **141** | N | 24 | 6518 | 6217 | 101792 | 0.0662 | 0.0630 | 1.0500 | 6 |
| **142** | N | 16 | 14831 | 14777 | 100000 | 0.1605 | 0.1599 | 1.0040 | 6 |
| **143** | N | 24 | 19797 | 19070 | 104368 | 0.2103 | 0.2018 | 1.0420 | 6 |
| **144** | N | 16 | 9202 | 8800 | 111576 | 0.0861 | 0.0822 | 1.0480 | 6 |
| **145** | N | 14 | 7141 | 6697 | 99011 | 0.0749 | 0.0700 | 1.0690 | 6 |
| **146** | N | 14 | 38389 | 37423 | 98718 | 0.4925 | 0.4766 | 1.0330 | 6 |
| **147** | N | 16 | 7696 | 7869 | 104465 | 0.0765 | 0.0783 | 0.9770 | 6 |
| **148** | N | 17 | 21755 | 21295 | 110643 | 0.2189 | 0.2138 | 1.0240 | 6 |
| **149** | N | 13 | 10077 | 10277 | 100554 | 0.1056 | 0.1078 | 0.9790 | 6 |
| **150** | N | 13 | 8956 | 8928 | 104216 | 0.0899 | 0.0896 | 1.0030 | 6 |
| **151** | N | 22 | 78623 | 78054 | 103239 | 1.4337 | 1.4108 | 1.0160 | 6 |
| **152** | N | 13 | 24056 | 24528 | 103826 | 0.2636 | 0.2695 | 0.9780 | 6 |
| **153** | N | 18 | 18875 | 18476 | 103541 | 0.2013 | 0.1966 | 1.0240 | 6 |
| **154** | N | 13 | 10560 | 10718 | 104929 | 0.1061 | 0.1077 | 0.9840 | 6 |
| **155** | N | 16 | 6921 | 7234 | 106163 | 0.0674 | 0.0706 | 0.9550 | 6 |
| **156** | N | 16 | 8588 | 8729 | 99698 | 0.0901 | 0.0916 | 0.9830 | 6 |
| **157** | N | 16 | 8128 | 8579 | 95927 | 0.0885 | 0.0937 | 0.9450 | 6 |
| **158** | N | 17 | 16195 | 15553 | 107557 | 0.1632 | 0.1562 | 1.0450 | 6 |
| **159** | N | 14 | 16254 | 16747 | 107875 | 0.1633 | 0.1687 | 0.9680 | 6 |
| **160** | T21 | 25 | 22018 | 20490 | 99058 | 0.2514 | 0.2317 | 1.0850 | 6 |
| **161** | N | 23 | 8144 | 7956 | 104612 | 0.0810 | 0.0791 | 1.0250 | 6 |
| **162** | N | 26 | 15565 | 15608 | 107055 | 0.1571 | 0.1576 | 0.9970 | 6 |
| **163** | N | 28 | 9231 | 9244 | 112421 | 0.0857 | 0.0858 | 0.9990 | 6 |
| **164** | N | 16 | 17433 | 16911 | 98426 | 0.1949 | 0.1885 | 1.0340 | 6 |
| **165** | N | 13 | 16256 | 16103 | 112518 | 0.1560 | 0.1545 | 1.0100 | 6 |
| **166** | N | 12 | 28034 | 27342 | 107743 | 0.3014 | 0.2927 | 1.0300 | 6 |
| **167** | N | 12 | 10226 | 10332 | 101997 | 0.1056 | 0.1068 | 0.9890 | 6 |
| **168** | N | 13 | 13836 | 13531 | 99117 | 0.1503 | 0.1468 | 1.0240 | 6 |
| **169** | N | 20 | 11401 | 11143 | 100407 | 0.1205 | 0.1176 | 1.0250 | 6 |
| **170** | T21 | 13 | 11309 | 10235 | 99714 | 0.1204 | 0.1083 | 1.1110 | 6 |
| **171** | N | 14 | 9086 | 9102 | 108419 | 0.0875 | 0.0877 | 0.9980 | 6 |
| **172** | N | 15 | 25715 | 26320 | 101913 | 0.2908 | 0.2988 | 0.9730 | 6 |
| **173** | N | 14 | 6847 | 6858 | 99052 | 0.0716 | 0.0717 | 0.9980 | 6 |
| **174** | N | 16 | 14256 | 14017 | 98264 | 0.1567 | 0.1539 | 1.0180 | 6 |
| **Sample** | **Fetal ploïdy** | **Gestational age (GW)** | **k 21** | **k ref** | **n** | **λ 21** | **λ ref** | **Chromosomal ratio (21/ref)** | **Number of replicates** |
| **175** | N | 12 | 20416 | 20513 | 106318 | 0.2132 | 0.2144 | 0.9950 | 6 |
| **176** | N | 17 | 17563 | 18210 | 104074 | 0.1848 | 0.1923 | 0.9610 | 6 |
| **177** | N | 19 | 17542 | 16971 | 72320 | 0.2778 | 0.2674 | 1.0390 | 6 |
| **178** | N | 16 | 11414 | 11424 | 101316 | 0.1195 | 0.1196 | 0.9990 | 6 |
| **179** | N | 13 | 10850 | 11110 | 105034 | 0.1090 | 0.1118 | 0.9750 | 6 |
| **180** | N | 16 | 12390 | 12217 | 103884 | 0.1270 | 0.1251 | 1.0150 | 6 |
| **181** | N | 18 | 13447 | 13285 | 107221 | 0.1340 | 0.1323 | 1.0130 | 6 |
| **182** | N | 13 | 9284 | 8979 | 100976 | 0.0964 | 0.0931 | 1.0360 | 6 |
| **183** | N | 14 | 11956 | 11561 | 102298 | 0.1243 | 0.1199 | 1.0360 | 6 |
| **184** | N | 23 | 8354 | 8239 | 62448 | 0.1436 | 0.1415 | 1.0150 | 6 |
| **185** | N | 32 | 56078 | 57284 | 96546 | 0.8695 | 0.8998 | 0.9660 | 6 |
| **186** | N | 22 | 12408 | 12714 | 103126 | 0.1282 | 0.1316 | 0.9740 | 6 |
| **187** | N | 17 | 18688 | 17890 | 92697 | 0.2251 | 0.2144 | 1.0500 | 6 |
| **188** | N | 13 | 9444 | 9225 | 105879 | 0.0934 | 0.0912 | 1.0250 | 6 |
| **189** | N | 16 | 13906 | 13759 | 101846 | 0.1468 | 0.1451 | 1.0120 | 6 |
| **190** | N | 16 | 12190 | 12027 | 108805 | 0.1188 | 0.1171 | 1.0140 | 6 |
| **191** | N | 13 | 6425 | 6674 | 92733 | 0.0718 | 0.0747 | 0.9610 | 6 |
| **192** | N | 14 | 6062 | 5972 | 102341 | 0.0611 | 0.0601 | 1.0160 | 6 |
| **193** | N | 13 | 11729 | 11541 | 95022 | 0.1317 | 0.1295 | 1.0170 | 6 |
| **194** | N | 24 | 12442 | 11890 | 100103 | 0.1327 | 0.1264 | 1.0500 | 6 |
| **195** | N | 13 | 9642 | 9290 | 103414 | 0.0979 | 0.0941 | 1.0400 | 6 |
| **196** | N | 16 | 14861 | 14643 | 107396 | 0.1489 | 0.1466 | 1.0160 | 6 |
| **197** | N | 13 | 14534 | 14320 | 100738 | 0.1558 | 0.1533 | 1.0160 | 6 |
| **198** | N | 18 | 8760 | 8468 | 106914 | 0.0855 | 0.0825 | 1.0360 | 6 |
| **199** | N | 29 | 12577 | 12744 | 99727 | 0.1348 | 0.1367 | 0.9860 | 6 |
| **200** | N | 12 | 7768 | 7839 | 102069 | 0.0792 | 0.0799 | 0.9910 | 6 |
| **201** | N | 27 | 14709 | 14960 | 101769 | 0.1561 | 0.1590 | 0.9820 | 6 |
| **202** | N | NA | 7683 | 7785 | 102932 | 0.0776 | 0.0786 | 0.9860 | 6 |
| **203** | N | 12 | 8548 | 8385 | 115797 | 0.0767 | 0.0752 | 1.0200 | 6 |
| **204** | N | 16 | 10688 | 10399 | 112449 | 0.0999 | 0.0970 | 1.0290 | 6 |
| **205** | N | 12 | 35761 | 35403 | 109879 | 0.3937 | 0.3889 | 1.0120 | 6 |
| **206** | N | 20 | 17617 | 16745 | 105911 | 0.1819 | 0.1721 | 1.0570 | 6 |
| **207** | N | 19 | 6997 | 6989 | 105129 | 0.0689 | 0.0688 | 1.0010 | 6 |
| **208** | N | 26 | 70986 | 70210 | 108236 | 1.0667 | 1.0460 | 1.0200 | 6 |
| **209** | N | 27 | 28725 | 29214 | 103929 | 0.3235 | 0.3300 | 0.9800 | 6 |
| **210** | N | 17 | 10207 | 10180 | 100473 | 0.1071 | 0.1068 | 1.0030 | 6 |
| **211** | N | 19 | 6429 | 6458 | 108520 | 0.0611 | 0.0614 | 0.9950 | 6 |
| **212** | N | 32 | 25105 | 24183 | 104072 | 0.2761 | 0.2644 | 1.0440 | 6 |
| **213** | N | 15 | 14322 | 13847 | 107241 | 0.1434 | 0.1383 | 1.0370 | 6 |
|  | **Mean** | 17 | 13448 | 13583 | 122247 | 0.1388 | 0.1407 | 0.9965 |  |
|  | **Median** | 16 | 10317 | 10399 | 108742 | 0.0976 | 0.1020 | 1.0040 |  |
|  | **Minimum** | 9 | 1393 | 1424 | 62448 | 0.0111 | 0.0106 | 0.8019 |  |
|  | **Maximum** | 37 | 83159 | 85214 | 255026 | 1.4337 | 1.4108 | 1.1592 |  |

NA: Not Available
