# Supplementary material for: Could Digital PCR Be an Alternative as a Non-Invasive Prenatal Test for Trisomy 21: A Proof of Concept Study
Source: PLoS One. 2016 May 11;11(5):e0155009. doi: 10.1371/journal.pone.0155009 (PMC4864235; doi:10.1371/journal.pone.0155009)
Supplement: S3 Table — We used a set of three FAM TaqMan® hydrolysis assays for the APP, BRWD1 and RUNX1 genes (Assays identities respectively: Hs01344980_cn, Hs03026207_cn and Hs05550012_cn; Life Technologies, Carlsbad, CA, US) and three VIC TaqMan® hydrolysis assays for the ASTN1, FAF1 and PUM1 genes (Assays identities respectively: Hs05795637_cn, Hs06521574_cn and Hs06604919_cn; Life Technologies, Carlsbad, CA, US). ddPCR protocol was the same as described in the Materials and Methods section. k 21 and k ref are the number of positive droplets for chromosome 21 and the reference chromosome, respectively. n is the total number of PCRs. λ 21 and λ ref are the estimated mean number of copies per droplet, according to the Poisson distribution. (DOCX) [file pone.0155009.s006.docx]

**Supplementary Table 3: Results of ddPCR on a set of plasma DNA using chromosome 1 as a reference chromosome**

| **Sample** | **Fetal ploïdy** | **Gestational age (GW)** | **k 21** | **k ref** | **n** | **λ 21** | **λ ref** | **Chromosomal ratio (21/ref)** | **Number of replicates** |
| --- | --- | --- | --- | --- | --- | --- | --- | --- | --- |
| **214** | **T21** | 18 | 11766 | 11076 | 102204 | 0.1223 | 0.1147 | **1.0663** | 6 |
| **215** | **T21** | 14 | 11479 | 10719 | 106849 | 0.1137 | 0.1057 | **1.0751** | 6 |
| **216** | **T21** | 24 | 16226 | 14178 | 95659 | 0.1859 | 0.1604 | **1.1587** | 6 |
| **217** | N | 16 | 24828 | 24382 | 149607 | 0.1815 | 0.1779 | 1.0201 | 6 |
| **218** | N | 16 | 22272 | 21843 | 137420 | 0.1768 | 0.1731 | 1.0215 | 6 |
| **219** | N | 14 | 9457 | 9468 | 100024 | 0.0993 | 0.0994 | 0.9988 | 6 |
| **220** | N | 17 | 18738 | 18442 | 88427 | 0.2381 | 0.2339 | 1.0181 | 6 |
| **221** | N | 27 | 9920 | 9569 | 105876 | 0.0984 | 0.0947 | 1.0385 | 6 |
| **222** | N | 29 | 8247 | 7824 | 102651 | 0.0838 | 0.0793 | 1.0564 | 6 |
| **223** | N | 14 | 11286 | 11361 | 100438 | 0.1192 | 0.1200 | 0.9930 | 6 |
| **224** | N | 17 | 9801 | 9667 | 98540 | 0.1048 | 0.1033 | 1.0146 | 6 |
| **225** | N | 21 | 13308 | 13112 | 99494 | 0.1436 | 0.1413 | 1.0161 | 6 |
| **226** | N | 16 | 7176 | 7035 | 94457 | 0.0790 | 0.0774 | 1.0209 | 6 |
| **227** | N | 15 | 6557 | 6278 | 92314 | 0.0737 | 0.0704 | 1.0461 | 6 |
| **228** | N | 19 | 4837 | 4680 | 104205 | 0.0475 | 0.0460 | 1.0344 | 6 |
| **229** | N | 16 | 8064 | 7998 | 111282 | 0.0752 | 0.0746 | 1.0086 | 6 |
| **230** | N | 14 | 6055 | 5825 | 101962 | 0.0612 | 0.0588 | 1.0407 | 6 |
| **231** | N | 16 | 4344 | 4128 | 100680 | 0.0441 | 0.0419 | 1.0535 | 6 |
| **232** | N | 16 | 5170 | 5021 | 106870 | 0.0496 | 0.0481 | 1.0304 | 6 |
| **233** | N | 23 | 9251 | 9965 | 102415 | 0.0947 | 0.1024 | 0.9248 | 6 |
